# “This is going to be a nice-to-know, not a need-to-know”: absence of concern – a qualitative study of risk perception in early detection of type 1 diabetes

**DOI:** 10.3389/fcdhc.2026.1925120

**Published:** 2026-10-02

**Authors:** Emilia Bjergskov Pedersen, Pernille Bech Flarup, Louise Norman Jespersen, Mette Due-Christensen, Julie Christine Antvorskov, Flemming Pociot, Dan Grabowski

**Affiliations:** 1 Department of Prevention, Health Promotion & Community Care, Copenhagen University Hospital – Steno Diabetes Center Copenhagen, Herlev, Denmark; 2 Translational Type 1 Diabetes Research, Department of Clinical Research, Steno Diabetes Center Copenhagen, Herlev, Denmark; 3 Department of Clinical Medicine, Faculty of Health and Medical Science, University of Copenhagen, Copenhagen, Denmark; 4 Department of Public Health, University of Southern Denmark, Odense, Denmark

**Keywords:** early detection, first-degree relatives, psychosocial aspect, qualitative research, risk perception, type 1 diabetes

## Abstract

**Background:**

Type 1 Diabetes (T1D) is a chronic and demanding condition. In recent years, the development of immunomodulatory therapies has increased interest in early detection programs, which may delay disease onset and enable psychological preparedness. However, such programs also introduce new psychosocial considerations. This study explores how first-degree relatives of individuals with T1D perceive and experience participation in an early detection program.

**Methods:**

Semi-structured interviews were conducted with 28 adults aged 18–42 years participating in a Danish early detection program for T1D (DiaUnion). Data were analyzed using reflexive thematic analysis to explore patterns related to risk perception and meaning-making.

**Results:**

The analysis identified three main themes. First, narrative understanding of diabetes based on parental experience illustrates how perceptions of risk are shaped by family narratives and lived experiences. Second, general understanding of diabetes shows that perceptions are often informed by personal beliefs rather than biomedical knowledge. Third, interpretation of the screening result demonstrates that results are frequently downplayed and assigned limited personal significance. Across themes, participants actively constructed meaning around their risk through coping strategies such as normalization, reframing, and emphasizing controllability, which contributed to a generally low perceived risk.

**Conclusion:**

Risk perception among first-degree relatives is shaped by pre-existing personal and familial narratives, which influence how screening results are interpreted. Early detection programs should therefore move beyond standardized communication and adopt more individualized approaches that account for participants’ existing frameworks of understanding and coping.

## 1 Introduction

Type 1 diabetes (T1D) is an autoimmune disease in which the body targets and destroys the insulin-producing β-cells located in the pancreatic islets of Langerhans. This irreversible process leads to an absolute deficiency of endogenous insulin and chronic hyperglycemia. Although T1D is most commonly diagnosed during childhood or adolescence, it can develop at any age, often with little or no warning (1).

Managing T1D requires continuous and demanding self-care throughout life. Individuals must monitor their blood glucose levels multiple times a day and administer insulin via multiple daily injections or an insulin pump. Glycemic levels are influenced by numerous lifestyle factors including physical activity, dietary intake, hormonal fluctuations, emotional stress, and illness (2). The need to constantly balance these factors makes living with T1D both complex and burdensome, posing significant challenges to maintaining optimal glucose levels and overall quality of life (3).

Over recent years, the ability to detect T1D early has emerged. The development of immunomodulatory therapies, such as teplizumab, which has shown an ability to delay the clinical onset of T1D, has intensified global interest in early detection and timely intervention (4–6).

Although T1D typically progresses silently over several years, it can now be identified in its preclinical stages through a blood test that detects multiple islet autoantibodies and early signs of dysglycemia. The presence of one autoantibody is associated with an approximately 15% risk of developing T1D within 10 years, and having two autoantibodies is associated with an almost 85% risk of T1D development within the next 15 years (7). Early detection offers medical advantages, such as delaying disease onset and reducing the risk of diabetic ketoacidosis and may provide individuals and families with time to prepare psychologically and practically for a potential life with diabetes (4). A negative screening result does not eliminate future risk but can be used as an indication of current risk.

All first-degree relatives of individuals with T1D carry a markedly increased lifetime risk of developing the condition, estimated at around 5–10%, compared to approximately 0.3% in the general population (1, 4). Despite this lifelong elevated risk, most early detection programs and research initiatives have traditionally focused on children, even though adults over the age of 18 account for more than half of all newly diagnosed T1D cases (8). This discrepancy highlights a significant gap in our understanding of how asymptomatic adults from high-risk families experience participation in early detection initiatives and how they navigate the psychosocial implications of a potential diagnosis (9).

Responses to increased disease risk are influenced not only by objective risk information but also by individuals’ beliefs about illness, perceived vulnerability, perceived control, and emotional reactions (10). The Common-Sense Model of Self-Regulation proposes that individuals construct personal representations of illness based on prior experiences, social influences, and available information, and that these representations shape coping responses and emotional adjustment (11). Complementary perspectives from health behavior theory further suggest that perceived susceptibility and perceived control may influence responses to disease risk (12).Within the context of T1D screening, these perspectives suggest that risk information is actively interpreted rather than passively received, and that individuals may engage in coping and meaning-making strategies such as normalization, cognitive reframing, and efforts to maintain a sense of control.

Emerging psychosocial research demonstrates that participation in T1D screening may evoke uncertainty regarding the meaning of screening results, concerns about future disease development, and questions related to coping, psychosocial support needs, and preparation for a potential diagnosis. Individuals identified as being at increased risk may experience distress, uncertainty, and shifts in self-perception even in the absence of symptoms, while risk perceptions are often shaped by lifestyle beliefs, family experiences, and cultural understandings rather than biomedical information alone (10, 13–15). At the same time, research suggests that individuals frequently underestimate their personal risk despite belonging to clinically elevated risk groups, whereas a family history of diabetes may increase feelings of vulnerability (16). Nevertheless, little is known about how adult first-degree relatives interpret, negotiate, and respond to risk information within contemporary early detection programs.

Across Europe, the EDENT1FI project has introduced screening activities with biomarkers for autoantibodies for children in multiple countries, while simultaneously strengthening existing programs in Denmark, Sweden, and Germany (17). (EDENT1FI, n.d.). The Danish–Swedish collaboration DiaUnion has established a cross-border screening infrastructure, which in Denmark is focused on first-degree relatives of individuals with T1D. Denmark stands out in this field as the only country screening first-degree relatives and not the general population. By combining autoantibody and genetic screening, the initiative has demonstrated the feasibility of large-scale recruitment through innovative approaches such as home-based blood sampling and digital follow-up systems (18). The present research aims to contribute knowledge that can inform the development of early detection programs that are not only clinically sound, but also ethically and emotionally attuned to the needs of the participants they serve.

## 2 Aims

The study explores the experiences of adult first-degree relatives (aged 17–42 years) participating in a T1D early detection program. Specifically, the study aims to answer the following research questions:

1. What are the emotional, psychological, and practical challenges associated with participation in a T1D early detection program?
2. How do adult first-degree relatives perceive and interpret their risk of developing T1D, and what support needs do they identify in relation to the healthcare system?

## 3 Methods

### 3.1 Setting

The present study was conducted in association with DiaUnion. In Denmark, the early detection process is presently limited to first-degree relatives of individuals with T1D aged 1–40 years, using either home-based test kits or blood samples collected at Steno Diabetes Center Copenhagen. The presence of two or more persistent islet autoantibodies is associated with an almost 100% risk of developing T1D later on (7, 19). In addition to diabetes-related islet autoantibodies, participants in DiaUnion are also tested for markers associated with celiac disease and autoimmune thyroid disorders (20).

The study examined two groups within DiaUnion: parents of children aged 1–17 who had undergone screening, and adults aged 18–42 who had been screened. While the study by Flarup et al. elaborates on the findings from the parent group, the present article focuses exclusively on the adult participants (21).

### 3.2 Design

The present study was designed as a qualitative, exploratory study investigating the psychosocial experiences of adult first-degree relatives of individuals with T1D who had undergone autoantibody screening to assess their own risk of developing diabetes. The study employed semi-structured individual interviews to explore adults’ experiences and reactions related to the early detection process. The interview guide included 16 questions along with follow-up prompts, which covered the following broad themes:

- Family Introduction
- Motivation & Experience of the Process
- Preparation, Information & Risk Perception
- Emotions, Thoughts & Concerns
- Family Dynamics
- Results & Follow-up
- General Reflections & Considerations

### 3.3 Recruitment procedure

The participants were randomly selected in Redcap based on the timing of the blood sample, the aim being to include families and adults at different stages of the process. Recruitment involved phone calls, voicemail messages, and text messages containing information about the study and the option to respond via the same channels.

The researchers applied no additional inclusion or exclusion criteria beyond participants’ involvement in the DiaUnion initiative. The goal was to recruit a balanced sample of individuals who had already received their results and those still awaiting them, ensuring a comprehensive understanding of the themes emerging throughout the process. The final sample consisted of 28 participants: 14 adults awaiting results (AW), seven adults who had received a negative result (AAN), and seven adults who had received a positive result indicating one or more autoantibodies (AAP). Furthermore, the researchers aimed to recruit enough participants who had tested positive to gain knowledge about the need for post-screening follow-up (**Table 1**).

**Table 1:** Participant overview.

Group	Number of participants
Potential informants contacted – total	87
Informants who agreed to participate – total	59
Parents of tested children (separate paper)	31
Adults	28

### 3.4 Data collection

Data collection took place from May 1 to August 15, 2025. Interviews were conducted either via telephone, through home visits or meetings at the clinic. In total, 19 telephone interviews and nine in-person interviews were conducted. The interviews lasted between 12 and 37 minutes, were audio-recorded, and transcribed verbatim. All interviews were conducted in Danish by PF. It should be noted that the proportion of participants with positive results is not representative of the underlying screening cohort.

To minimize participant burden, telephone interviews were offered as an option. This format was chosen by some participants primarily for pragmatic and geographic reasons. It allowed for flexibility in scheduling and enabled participation from participants’ own homes, which was considered particularly relevant given the potentially sensitive nature of the study topic. When feasible, face-to-face interviews were conducted based on participant preference.

No systematic differences were observed between telephone and in-person interviews in terms of interview length, depth of reflection, or the themes identified. Participants in both formats appeared equally able to articulate their experiences. While in-person interviews allowed for the observation of non-verbal cues, telephone interviews may, in some cases, have facilitated openness by providing a greater sense of privacy and anonymity. Both formats were therefore considered methodologically appropriate for the study aims.

### 3.5 Ethics

Ethical considerations become especially critical in early detection programs, as the process may introduce uncertainty and vulnerability for participants.

The study was assessed by the Ethics Committee of the Capital Region of Denmark (journal number F-25026891). The study complies with the ethical guidelines of the Declaration of Helsinki (22) and was approved by the Danish Regional Data Protection Agency.

All participants were contacted prior to the interview and informed about the purpose of the study, what participation would entail, and that participation was entirely voluntary. Participants who expressed interest were subsequently provided with written information about the study. All participants gave informed consent prior to the interview.

The preventive treatment teplizumab was approved by the European Medicines Agency (EMA) on January 8th 2026. However, at the time interviews were conducted for this study, no preventive treatments had yet received approval (23). To address the ethical challenges, we ensured that participants, particularly those receiving positive results, had access to support from healthcare professionals before, during, and after the interview process. Interviews were conducted with sensitivity to and respect for participants’ emotional responses, acknowledging the vulnerability and complexity involved in navigating early detection in the absence of available and approved preventive treatments.

### 3.6 Analytical approach

The data were analyzed using reflexive thematic analysis, allowing for an inductive exploration of recurring patterns and meanings related to participants’ psychosocial experiences (24). Each phase of the analysis was discussed among the authors to ensure that consensus was reached regarding the main themes. In Phase 1, the transcripts were listened to and re-read to gain an overall impression of the data. In Phase 2, author EBP developed initial codes using the qualitative data analysis program NVIVO to organize the data. In Phase 3, the authors explored the relationship between the codes and sorted them into potential themes. In Phase 4, the authors evaluated and refined the themes, scrutinizing the data underlying them to ascertain whether they exhibited a consistent pattern. In Phase 5, the final themes were defined and named.

## 4 Findings

Characteristics of participants are presented in **Table 2**.

**Table 2:** Baseline characteristics of the study participants (N = 28).

Characteristic	n (%)
Gender	
Men	9 (32.1)
Women	19 (67.9)
Age group (years)	
17-25	13 (46.3)
26-34	10 (35.7)
35-42	5 (17.9)
Family history of T1D	
Mother	8 (28.6)
Father	13 (46.4)
Sibling	5 (17.9)
Other family member	2 (7.1)
Antibody status	
Adult Waiting (AW)	14 (50.0)
Adult Answer Negative (AAN)	7 (25.0)
Adult Answer Positive (AAP)	7 (25.0)
Educational status	
Long-cycle higher education	16 (57.1)
Medium-cycle higher education	6 (21.4)
Vocational/upper secondary education	4 (14.3)
Primary and lower secondary education	2 (7.1)
Marital status	
Married	4 (14.3)
Cohabiting	15 (53.6)
Lives alone but has partner	2 (7.1)
Single	6 (21.4)
Unknown	1 (3.6)
Place of residence	
Urban area	23 (82.1)
Suburban area	3 (10.7)
Rural area	2 (7.1)
Ethnic background	
Caucasian	28 (100)
Other	0 (0)

When presenting the results, each quote is labelled with a code indicating whether the participant has tested positive, negative or is still waiting for the results. The labels are: “AAP” = *Adult Answer Positive*, “AAN” = *Adult Answer negative* or “AW” = *Adult Waiting*.

To understand how adult first-degree relatives perceive and experience participation in an early detection program, the analytical focus was on how first-degree relatives make sense of their own risk of developing T1D. To describe this aspect of their experience in depth, we developed three themes: 1) narrative understanding of diabetes shaped by parental experience, 2) general understanding of diabetes, and 3) interpretation of the screening result. Together, these themes illustrate how the first-degree relatives construct meaning around their personal risk and how this meaning-making process influences the worries, uncertainties, and emotional responses that accompany participation in an early detection study.

### 4.1 Theme 1: narrative understanding of diabetes based on parental experience

One main finding concerns how knowledge about diabetes, gained from parents, shapes how first-degree relatives experience their own risk. Those whose parents have been open about their diabetes throughout childhood tend to view the condition as a normal and integrated part of everyday life. For these individuals, diabetes is not associated with fear or uncertainty but rather with familiarity.

#### AAN3

*“(…) I honestly don’t think I’ve thought that much about it, because I feel that talking about diabetes has become very normalized in my family. My mother doesn’t do finger pricks anymore. She now has one of those pump sensors. But I can remember that she used to prick her finger, and we also thought it was kind of fun to measure our own blood sugar. So we were part of that whole process as well. Because of that, I don’t think I ever felt that having diabetes was the end of the world. Not at any point. I think I’ve actually been quite accepting and calm about it, and I haven’t been very worried about the outcome, whatever it might be.”*

A consistent pattern shows how first-degree relatives whose parents openly discussed and managed their diabetes tend to express calmness about the possibility of developing T1D themselves. However, this openness appears to be selective. The first-degree relatives describe positive experiences, while rarely mentioning episodes of severe complications. This suggests that some parents may have practiced a form of protective disclosure, shielding their children from the more difficult aspects of the disease. As a result, the first-degree relatives may not have a full picture of what living with T1D entails. Their understanding is shaped by a narrative in which diabetes is controllable and relatively uncomplicated. This has both positive and negative implications. The narrative may contribute to an underestimation of the seriousness of the disease. Across this population, statements like *“it’s just diabetes”* or *“it could be something far worse”* illustrate a recurring pattern in which T1D is perceived as a relatively minor condition. On the other hand, the decision of not involving their children in the negative sides of diabetes seems to be a decision made by parents to protect their children from unnecessary worry. Knowing you have a risk of developing an illness you have seen as very negative may take up a great deal of mental space and may limit how you live your life due to fear of developing it.

#### AAN2

*“(…) Diabetes has taken up an enormous amount of space in my sister’s and my life, and in my mother’s life as well - really a lot. Our father was very bad at managing it and often had low blood sugar episodes and things like that. So there has been illness that has frightened us, because sometimes it was really difficult. That meant we experienced diabetes from a very negative side, and it made you want, for the love of God, not to do anything that might cause you to get it.”*

Many of the first-degree relatives have had traumatic experiences throughout their childhood with a relative whose diabetes was not well-regulated, which has instilled the narrative that diabetes is a disease with many negative side-effects. The thought of getting diabetes has therefore always been an underlying part of their mental awareness, resulting in them paying extra attention to what things in life might increase or decrease chances of developing it. Generally, the first-degree relatives who have experienced diabetes as something hard to manage are more worried.

The narrative the parents have given shapes the way they see the disease. This narrative is formed through how the parents have handled diabetes themselves and their own narratives. This is emphasized by AW14 whose father has T1D:

#### AW14

*“(…) In his family it had always been a taboo that he had this condition. It was something that was somewhat hidden that he had diabetes. I’m not exactly sure why, but it had always been that way with my grandparents. He has also had some difficulties managing his diabetes and has experienced many of these insulin shocks. So I think that also made him feel that it was a good thing that we signed up.”*

When diabetes is described as something that has been hidden or treated as a taboo within families, the condition is understood not only as a physical illness, but also as something associated with shame or stigma. First-degree relatives describe how this concealment has been connected to difficulties in managing the disease, resulting in complications such as insulin shocks. The data shows that parents who have had challenging experiences with diabetes, concerning not only management, but also the social meanings attached to the condition, often express a desire to prevent their children from having similar experiences. Encouraging participation in early detection can therefore be understood as an attempt to break with a family narrative in which diabetes is associated with shame.

Another way the parents’ narrative on diabetes affects the first-degree relative’s narrative concerns the temporality of the disease. Multiple relatives mention that their parents got diabetes at a specific age and, therefore, they perceive that they are or are not at risk of developing it themselves.

#### AW2

*“(…) Because I know that it can be hereditary, and my mother got it after my grandmother. So even if the risk may be low, it was still something that happened. And my mother got it at the age of 33, so that’s also relevant for me right now, because I’m turning 32 next month. So it also fits right into the timing.”*

A temporal understanding of risk is common in this population, where individuals often refer to their parents’ early onset of diabetes and conclude that, because they are now adults, they have passed the period in which they consider themselves vulnerable. This misunderstanding may be because of the parents’ narrative, but also because general knowledge about diabetes might be lacking, even among first-degree relatives.

### 4.2 Theme 2: general understanding of diabetes

Among the first-degree relatives, T1D is often discussed in terms of familiar risk factors, symptoms, and complications, yet these understandings tend to be partial or imprecise. While it might be assumed that adults with a close family member with T1D have substantial knowledge about the condition, their accounts do not always reflect this.

#### AW13

*“I actually haven’t thought it was particularly likely [to test positive]. I don’t know why, I’ve always thought that type 1 is something that just occurs kind of randomly. You don’t really know why. (…) In my mind, type 2 is more of a hereditary thing, because it’s also influenced by lifestyle, or certain lifestyle-related conditions, which can also … In my head, it has seemed more likely that there might be a connection there.”*

While T2D is indeed influenced by lifestyle factors, this distinction appears to lead some first-degree relatives to underestimate the relevance of hereditary risk in T1D. Multiple first-degree relatives emphasize that they are highly conscious of maintaining a healthy lifestyle through diet and exercise in an attempt to prevent developing T1D. This indicates that some of them view lifestyle as a meaningful way to influence their risk, even though their understanding of the disease is shaped more by personal beliefs and experiences than by biomedical knowledge.

#### AAP4

*“I’m actually quite calm, because I feel that I live a healthy lifestyle. And I don’t know how much it matters, but based on what I’ve understood, it makes a big difference to eat properly and live relatively healthily. So in that way, it has made me feel calm, but I don’t know if it’s a kind of false sense of security.”*

The first-degree relatives both rely on and question the effectiveness of various preventive strategies. Across accounts from this population, there is a clear sense of wanting to take action to counter the risk associated with having a close family member with T1D. When faced with a potential future life with T1D, adopting healthy habits may function as a coping strategy - an attempt to regain control in a situation defined by uncertainty. A false sense of security may therefore be related not only to the belief that lifestyle can prevent T1D, but also to the emotional comfort of feeling proactive rather than passive. Engaging in these behaviors allows first-degree relatives to resist the idea of inevitability and maintain a sense of control. While healthy lifestyle behaviors may provide practical and psychological benefits, including supporting overall health and potentially facilitating adaptation should T1D develop, there is currently no evidence that such behaviors prevent the onset of T1D.

Furthermore, when looking at the general level of knowledge among first-degree relatives, many describe T1D as not particularly serious. They often portray it as manageable and contrast it with other conditions they perceive as far more severe.

#### AAN5

*“(…) But then again, it’s not something you couldn’t live with, it’s not that kind of severe disease. Of course, diabetes is serious in a way, but there are also many treatment options with medication and so on.”*

Here, diabetes is framed as a condition that can be controlled due to the availability of treatment options. This wording is interesting, as diabetes is a chronic disease without a cure, and therefore not something that can be treated in the traditional sense. What exists are management strategies that allow individuals to live with the disease (1). The choice of words may therefore reflect the broader assumption that medical advancements have progressed to a point where diabetes is highly manageable, and perhaps even close to being curable. This perception appears to reduce the fear of developing T1D, as it is imagined to have minimal impact on their daily lives.

### 4.3 Theme 3: interpretation of the screening result

Across accounts from the first-degree relatives, a consistent theme was identified around their interpretation of the screening result and their overall understanding of the early detection program.

#### AW1

*“Well, I can say that I know a few people who have it [Diabetes], and they live with it just fine. Without any major issues, of course there are some things you have to be mindful of and so on. But otherwise, they live normal lives, so it’s not something I think of as being like getting cancer or anything like that. That’s why it doesn’t worry me that much. I feel like there’s a pretty good understanding of what to do if you were to get it. There are things you can do, and the doctors know how to handle it”*

The first-degree relatives often draw on the experiences of people they know who live well with diabetes and compare T1D to conditions they perceive as far more severe, such as cancer. Through this comparative framing, diabetes is placed in a category of illnesses understood as manageable rather than life-threatening. Several first-degree relatives employ this logic, emphasizing that “it could be worse” and thereby positioning T1D as a relatively minor condition. On an ultimate level, they are comparing diabetes to death itself, making any chronic illness appear less significant simply because it is survivable. This comparison, however, does not necessarily translate meaningfully into everyday life and may unintentionally downplay some of the challenges associated with living with T1D. Viewed through the lens of cognitive reframing, this minimization may function as a coping strategy. By focusing on the idea that others have it worse, first-degree relatives create psychological distance from the underlying fear associated with their own risk. This reframing makes the possibility of developing T1D seem less threatening. In the quote it is also emphasized that “there are things you can do” and that doctors “know how to handle it.” This might reflect the perception that diabetes management is more medical advanced than it actually is. Consequently, the complexity and daily demands of living with T1D may be underestimated.

In that regard, some first-degree relatives also mention that the early detection result is not really important to them.

#### AW4

*“(…) But I think it’s more like this is something that’s nice to know, it’s not something you need to know. We can live just as good a life without knowing this as we could if we knew it.”*

In many narratives, early detection is framed as useful but not necessary, indicating that risk information does not consistently hold strong personal meaning. For these relatives, the screening result appears to have little practical or emotional significance. Knowing their risk does not seem to change how they live their everyday lives. In this sense, the result is experienced as largely inconsequential, and the distinction between knowing and not knowing becomes minimal. At the same time, this position appears somewhat paradoxical, as these first-degree relatives have nevertheless chosen to take part in the early detection program. This points to an underlying ambivalence in how risk knowledge is understood. While they engage in the act of screening, the data shows they simultaneously distance themselves from the potential emotional implications of the result. Downplaying the importance of the result functions as a way of managing uncertainty. By framing the information as non-essential, the first-degree relatives reduce the need for emotional preparation and avoid confronting the possible consequences of a positive result. In this way, the stance of “it doesn’t really matter whether I know or not” can be understood as a coping strategy rather than a genuine lack of concern.

This pattern might also reflect a limited understanding of what the screening result entails. The first-degree relatives often interpret results as a general or low-level indication of risk rather than a near certainty. This misinterpretation may reinforce the perception that the result is not particularly important, either because the seriousness is underestimated or because the implications are not fully understood. This is illustrated by AAP6 who has tested positive for one autoantibody but is unsure what that means for her.

#### AAP6

*“I was told that it’s one of those that aren’t really that serious. I’m still not really in what you’d call the danger zone, I think. But that I should pay attention to this. The way I understand it, it mostly just means that I have this antibody, and they’re aware of it now. I’m not in the risk group yet, but I’m kind of in a group that could become a risk group. That’s how I understand it. I’m in a category where something could develop, but that doesn’t mean it will.”*

The first-degree relatives who test positive for one autoantibody often express a sense of nonchalance. They describe the result as not serious and position themselves outside what they perceive as the real risk group. This may stem from a misunderstanding of the information provided, but it may also reflect how the research staff communicate the meaning of one autoantibody. Several first-degree relatives report similar interpretations, suggesting that the message about risk is not consistently understood. A broader pattern emerges in which first-degree relatives who receive a single autoantibody result tend to minimize its importance. Several describe the result as insufficient to trigger concern or a need for support, noting that their response would likely be different if multiple autoantibodies were involved. This indicates that a small risk is not perceived as threatening.

Many of the first-degree relatives have lived with an underlying sense of risk their entire lives. Receiving a positive result may therefore feel more like a confirmation of something already expected than a new source of concern. Another interpretation is that this downplaying functions as a form of defensive coping. By framing one autoantibody as not serious, first-degree relatives create a narrative in which the result becomes manageable. This aligns with the broader “it could be worse” logic identified earlier. By comparing their situation to more severe outcomes, first-degree relatives minimize the psychological impact of the result and maintain a sense of control.

Another tendency is how first-degree relatives who tested negative say that the result did not matter to them as illustrated by AAN3:

#### AAN3

*“Yes, I had kind of forgotten about it [the test result]. I was a bit excited when I saw that I had received something in my e-Boks [digital mailbox]. I was like, “oh, exciting, “ because it could have gone either way. But it wasn’t like I was thinking, “oh, thank God it was negative.” I was just like, “well, now I know”“.*

This narrative points to a common pattern where the first-degree relatives do not dwell on the test while awaiting the result. For several of them, the test fades into the background, with some even forgetting about it until the result arrived. This suggests that the screening does not generate ongoing worry, as the level of concern is so low that the first-degree relatives forget they are part of an early detection program. For many, diabetes is perceived as a familiar and manageable condition rather than a life-altering diagnosis. The quote also states that, while receiving a negative result was nice, the reaction would probably not have been significantly different had the result been positive. This further indicates that the presence or absence of risk is not experienced as emotionally consequential. The disease is seen as something one can live with, and therefore the negative result does not produce the relief one might expect.

The first-degree relatives often interpret the result as a form of reassurance, at times treating it as a definitive outcome, both for themselves and regarding the perceived risk of passing on a genetic predisposition to their children, as seen in the following quote:

#### AAN5

*“If I had tested positive for something, then there might also have been something I had passed on to my children. So in that way, I think it gives me a bit of reassurance to find out that there’s nothing. Because then you also haven’t passed anything on, so to speak.”*

This suggests that some first-degree relatives release an underlying worry based on a false sense of certainty.

It is uncertain whether this misunderstanding reflects gaps in communication or whether first-degree relatives actively welcome the opportunity to feel safe, even if that safety is provisional. The overall finding, however, is that negative results are often treated as conclusive, although the screening offers only a point-in-time indication rather than a comprehensive evaluation of long-term risk.

## 5 Discussion

The present findings illustrate how adult first-degree relatives construct and negotiate their perception of risk within the context of an early detection program for T1D. Risk perception is shaped not only by parental knowledge and general understanding of diabetes, but also by the personal narratives participants have developed throughout their lives. These narratives appear to function as coping mechanisms that help first-degree relatives maintain psychological stability in the face of an uncertain future risk. Rather than reflecting a lack of understanding, they may represent active efforts to make risk more manageable and less disruptive to everyday life.

Our study contributes new insights into how adult first-degree relatives make sense of their risk of developing T1D. While existing literature often describes this group as experiencing heightened vulnerability, uncertainty, and distress when confronted with elevated risk, our findings challenge this assumption (13, 16). Risk, in our study, is consistently downplayed, normalized, or reframed as non-threatening, even among those who tested positive for autoantibodies.

This discrepancy appears to stem from the narratives first-degree relatives have developed throughout their life, growing up with T1D in their immediate family. T1D is described as normal, manageable and not that serious by First-degree relatives, often because their parents intentionally or unintentionally shielded them from the more difficult aspects of the disease. This is in contrast with the assumption that lived experience increases risk awareness (16). Instead, our data suggest that familiarity with the disease reduce the worry of being a first-degree relative. Furthermore, previous findings have stressed that early detection aims to reduce uncertainty and prepare individuals psychologically (4). Yet our findings indicate that many first-degree relatives do not perceive uncertainty to begin with. Their general understanding of T1D, sometimes confused with lifestyle-related T2D, contributes to a sense of control and a belief that lifestyle can meaningfully reduce risk - downplaying their actual risk. Finally, the way first-degree relatives interpreted their screening results reveals a significant gap between clinical and personal meaning. As shown in the findings, both negative and single-autoantibody results are often interpreted as inconsequential or irrelevant. This is in contrast with the clinical significance of autoantibodies and the need for structured follow up (4, 9). The tendency to minimize results, even when risk is objectively elevated, suggests that early detection programs must consider not only how information is delivered, but also how individuals’ pre-existing narratives shape their understanding.

One central finding of the present study is that risk perception among first-degree relatives is shaped by family narratives that normalize T1D and reduce its perceived severity. Growing up in a family where diabetes is openly discussed and integrated into everyday life appears to foster familiarity rather than fear. Rather than being experienced as a threatening condition, T1D is understood as manageable. This finding aligns with the Common-Sense Model of Self-Regulation, which suggests that individuals develop personal representations of illness based on prior experiences and social context. In the present study, family narratives appeared to shape these illness representations, influencing both perceived severity and perceived vulnerability.

This normalization can be further understood in relation to selective disclosure within families. The findings suggest that while diabetes is openly present, the more burdensome aspects of the disease are often downplayed. As a result, first-degree relatives may develop an incomplete understanding of the condition, in which T1D is perceived as relatively uncomplicated to manage. This challenges the assumption that lived experience with the disease necessarily leads to increased risk awareness (16). Instead, familiarity appears to reduce, rather than heighten, perceived risk. This process may reflect a form of normalization-based coping, in which repeated exposure to a health condition reduces its perceived threat. By integrating diabetes into a narrative of everyday family life, first-degree relatives may be able to maintain a sense of normality and avoid viewing themselves as future patients.

Importantly, this narrative is not uniform. In cases where first-degree relatives were exposed to more poorly managed diabetes, risk is perceived as significantly more prominent. These experiences appear to disrupt the otherwise normalized understanding of the disease and introduce fear. This suggests that risk perception is not simply low or high but relies on the nature of lived experience.

While family narratives were not always explicitly described by participants in the present study, they appeared embedded within accounts of childhood experiences, family communication, and observations of how T1D was managed within the household. In contrast to our previous study, where family narratives were articulated more directly (21), their influence in the present material was often indirect, shaping illness representations, perceptions of susceptibility, and strategies for coping with risk. This suggests that family narratives may continue to influence risk perception even when they are no longer consciously recognized as narratives by participants themselves.

Furthermore, the findings highlight a temporal dimension of risk perception showing that first-degree relatives often relate their own perceived vulnerability to the age at which their parent was diagnosed, indicating that risk is understood as something that unfolds within a specific life window. As individuals move beyond this perceived period of vulnerability, their sense of risk may decrease. This temporal framing of risk suggests that individuals do not interpret genetic susceptibility as constant, but as something that fluctuates across life. Risk information is therefore not received in a neutral way but is filtered through an already existing understanding that may limit its impact. This temporal framing may also serve as a protective psychological function. If risk is perceived as belonging to a specific period of life, individuals who have moved beyond that period can distance themselves from feelings of vulnerability and uncertainty.

Another key finding of the present study is that first-degree relatives create psychological distance to their risk of developing T1D by reframing it as something that can be managed. A recuring strategy in this process of creating distance is the perceived emphasis on lifestyle, where relatives describe healthy lifestyle choices as a way to actively reduce their risk. While such practices may have limited relevance for the biomedical development of T1D, they appear to provide a sense of control in an otherwise uncertain setting. Perceived controllability is a central element in how individuals cope with health-related uncertainty. By focusing on behaviors they can influence, first-degree relatives may transform an uncontrollable genetic risk into something that feels more predictable and manageable. The emphasis on lifestyle therefore appears to represent not only a belief about disease prevention but also an attempt to maintain agency in the face of uncertainty. This pattern is also consistent with assumptions of the Health Belief Model, where perceptions of control and susceptibility influence responses to health threats. Participants’ emphasis on lifestyle may therefore reflect an attempt to increase perceived control despite limited influence on the biological development of T1D. However, this also introduces a false sense of security, where perceived control does not align with actual disease mechanisms.

In addition to this, T1D is frequently framed as a manageable condition, further contributing to the downplaying of both risk and potential consequences. Advances in treatment technologies and everyday familiarity with the disease appear to shape the expectation that T1D can be easily managed, and in some cases even understood as close to curable. This perception stands in contrast to clinical accounts of T1D as a condition requiring continuous self-management and significant emotional and cognitive effort (3, 9, 25).

These findings suggest that first-degree relatives downplay their risk of developing T1D and reframe the disease itself in ways that reduce its perceived impact. This aligns partially with existing literature, which highlights a tendency for adults to downplay their risk (16). However, it also contradicts previous findings suggesting that first-degree relatives experience heightened vulnerability (16). Rather than experiencing persistent worry, our findings indicate that first-degree relatives appear to actively construct a sense of control and reassurance, even when they are at elevated risk.

A final key finding of the present study concerns how screening results are interpreted and integrated into first-degree relatives’ understanding of their own risk. Across accounts, the results appear to hold limited personal and emotional significance. Rather than increasing levels of concern, screening outcomes are frequently downplayed. This suggests that biomedical risk information does not automatically translate into perceived personal relevance.

One explanation for this pattern may lie in the fact that, for adult first-degree relatives, risk is not experienced as new information but as a pre-existing condition of life. Having grown up with an awareness of familial risk, first-degree relatives have already developed cognitive and emotional strategies to manage this uncertainty. As a result, receiving a positive result does not necessarily introduce a new layer of concern, but rather confirms an already familiar understanding of being at risk. This may help explain why responses to positive results remain relatively muted. From this perspective, minimization of screening results may not simply reflect misunderstanding but an active effort to preserve existing narratives of low vulnerability. Integrating positive results into an already familiar understanding of risk may be less psychologically disruptive than reconstructing one’s self-understanding around the possibility of future disease.

At the same time, first-degree relatives actively engage in cognitive reframing strategies that minimize the perceived seriousness of both the disease and the result. Comparisons to other conditions, particularly cancer, illustrate how T1D is positioned as a manageable and non-life-threatening illness. While such comparisons may provide psychological relief, they also contribute to a downplaying of the long-term demands and consequences of living with T1D. Such comparisons can be understood as a form of cognitive reframing, whereby the perceived threat of one condition is reduced through comparison with illnesses considered more severe.

A further dimension of this finding is the apparent disconnect between clinical risk categories and interpretations of the results. Autoantibody status, which has significant predictive value in biomedical terms, is often understood in vague ways. Single autoantibody results are frequently interpreted as low-risk or irrelevant, while negative results are sometimes taken as definitive reassurance, despite representing only a temporary absence of markers. This indicates that the meaning of screening outcomes is not simply misunderstood but actively reshaped to fit existing narratives of control and low vulnerability.

This pattern raises important questions regarding the role of communication within early detection programs. The tendency to downplay results should therefore not be understood solely as a failure of knowledge transmission, but as part of a broader process of meaning-making.

An important consideration is that participants were interviewed at different stages of the screening process. Although participants differed with regard to antibody status and stage of the screening process, the overarching themes remained consistent across groups. This finding may indicate that long-standing family narratives and lived experiences with T1D exert a stronger influence on risk perception than the screening result itself. In this sense, screening outcomes appear to be incorporated into pre-existing understandings of risk rather than fundamentally reshaping them. Nevertheless, future studies specifically designed to compare responses across antibody-status groups may provide further insight into how risk information is interpreted over time.

Finally, the coexistence of participation in screening and the downplaying of its importance highlights an underlying ambivalence in how risk is approached. First-degree relatives actively seek out risk information yet simultaneously minimize its implications. This suggests that screening participation may serve functions beyond risk management alone, such as reassurance, curiosity, or a desire for control, rather than reflecting a perception of vulnerability.

We previously studied parents of children aged 1–17 years undergoing early screening (21). The findings from that study indicate that participation in screening can both provoke worry and provide temporary reassurance. For these families, early detection appears to foster a form of resilience, enabling parents and children to mentally prepare for a potential future diagnosis.

In contrast, the present findings suggest a markedly different pattern among adult first-degree relatives. While early detection is often assumed to facilitate psychological preparedness (4), adult first-degree relatives did not express a strong need to prepare for a potential diagnosis. Rather than representing new or disruptive information, risk was seen as an already integrated aspect of life.

These contrasting findings may be understood in relation to differences in experiences of the disease. Parents of children at risk often have direct, embodied experience with T1D, including its daily burdens. Their concerns are therefore oriented toward the future well-being of their child, and screening becomes a way to anticipate and prepare for what they perceive as a potentially challenging trajectory. In contrast, adult first-degree relatives have typically been exposed to the disease observationally rather than experientially. While they are familiar with T1D, they have not lived with it themselves, which may contribute to a less immediate or threatening perception of risk.

From this perspective, the differences between the two groups may reflect distinct forms of coping. Whereas parents appear to engage with screening as a mean of managing fear and preparing for a possible diagnosis, adult first-degree relatives seem to rely more on strategies that normalize and downplay their risk. This suggests that early detection is interpreted differently depending on one’s position in relation to the disease, highlighting the importance of tailoring communication and support within screening programs to the specific experiential context of participants.

Future screening programs targeting chronic diseases should take these findings into account, particularly the importance of clear communication regarding both the meaning of screening results and the associated risks. However, our findings also suggest that risk communication cannot be standardized alone, as individuals interpret information through pre-existing personal and familial narratives.

First-degree relatives in the present study actively constructed meaning based on prior knowledge and lived experience, indicating that screening programs are introduced into an already established interpretive framework. This highlights the potential value of moving beyond standardized communication strategies toward more individualized approaches that acknowledge and engage with individuals’ existing risk narratives.

In addition, it is important to recognize that risk information is not simply received and understood as intended. Rather, it is actively interpreted to align with existing beliefs and coping strategies. This suggests a need for increased attention to the gap between biomedical risk classification and personal risk perception. Bridging this gap may require new approaches to communication that support a more accurate and meaningful understanding of risk.

Furthermore, early detection programs should not assume that providing risk information will automatically lead to increased awareness or behavioral change. Instead, they must consider the contextual and experiential background in relation to which individuals interpret their risk and engagement with screening.

Finally, one important implication of the present study is that the need for psychosocial support may not always be explicitly expressed. The apparent absence of expressed need should therefore not automatically be interpreted as an absence of psychological burden. Rather, it may indicate that this burden is managed through normalization, cognitive reframing, perceived control, and other coping strategies that make concerns less visible in participants’ accounts. This suggests that support within screening programs should not rely solely on stated need, but rather proactively address the underlying processes of coping and meaning making.

## 6 Strengths and limitations in the study

One key strength of our study is that it, to the best of our knowledge, is one of the first qualitative investigations into the psychosocial dimensions of early detection in first-degree relatives. As such, it contributes novel and empirically grounded perspectives to a field in which qualitative evidence remains scarce. The study specifically adds new insights into how first-degree relatives make sense of risk, challenging existing literature on the topic.

Another strength of the study lies in its rich qualitative dataset, comprising 28 interviews conducted with first-degree relatives. By including participants with different experiences of T1D, our study provides a nuanced understanding of how first-degree relatives navigate the early detection process.

Still, several potential limitations should be considered when interpreting our findings.

First, the participant sample may not fully reflect the broader population eligible for early detection. Some individuals may have overlooked the digital invitation or were not reached through other recruitment channels, while others may have actively declined participation. Consequently, perspectives related to non-participation - particularly motivations and concerns - are not captured in the present study.

Second, first-degree relatives from ethnic minority backgrounds are underrepresented in the sample. This is particularly concerning given that ethnic minorities seem to be at increased risk of diabetic ketoacidosis at diagnosis (26). This limitation requires attention moving forward, as failure to ensure that early detection initiatives reach families with an ethnic minority background could exacerbate existing health inequalities.

## 7 Conclusion

The present study aimed to explore the perceived risk and experience of participation in early detection. Adult first-degree relatives generally report a low perceived risk of developing T1D, and participation in early detection does not substantially alter this perception. Although first-degree relatives do not explicitly express a need for support from the healthcare system, they continuously engage in coping strategies such as normalization, reframing, and downplaying to manage the underlying awareness of being at increased risk.

These findings challenge the assumption that risk information inherently increases awareness and psychological preparedness. Instead, our findings indicate that risk is interpreted through pre-existing personal and familial narratives that fundamentally shape its meaning and impact.

Furthermore, our results show that risk perception is not uniformly high or low, but contingent on individual experiences with the disease. Familiarity with T1D contributes to its reinterpretation as a manageable condition, allowing individuals to create psychological distance from its potential burdens. Within this framework, positive screening results are often experienced not as new or disruptive information, but as confirmation of an already established sense of vulnerability.

Our findings suggest that early detection programs may benefit from moving beyond standardized communication strategies toward more individualized approaches that take participants’ pre-existing narratives into account.

## Data Availability

The interview data are not publicly available due to ethical and privacy considerations but may be available from the corresponding author upon reasonable request.
